# Spatial structuring of a *Legionella pneumophila* population within the water system of a large occupational building

**DOI:** 10.1099/mgen.0.000226

**Published:** 2018-10-12

**Authors:** Sophia David, Massimo Mentasti, Sandra Lai, Lalita Vaghji, Derren Ready, Victoria J. Chalker, Julian Parkhill

**Affiliations:** ^1^​ Pathogen Genomics, Wellcome Sanger Institute, Wellcome Genome Campus, Hinxton, Cambridge, UK; ^2^​ Respiratory and Vaccine Preventable Bacteria Reference Unit, Public Health England, Colindale, London, UK; ^3^​ Food, Water and Environmental Laboratory, Public Health England, Colindale, London, UK; ^†^​ Present address: The Centre for Genomic Pathogen Surveillance, Wellcome Genome Campus, Cambridge, UK.; ^‡^​ Present address: Microbiology Cardiff, Public Health Wales, University Hospital of Wales, Cardiff, UK.

**Keywords:** *Legionella pneumophila*, Legionnaires' disease, whole-genome sequencing, genomic diversity, water system, occupational building

## Abstract

The diversity of *Legionella pneumophila* populations within single water systems is not well understood, particularly in those unassociated with cases of Legionnaires’ disease. Here, we performed genomic analysis of 235 *L. pneumophila* isolates obtained from 28 water samples in 13 locations within a large occupational building. Despite regular treatment, the water system of this building is thought to have been colonized by *L. pneumophila* for at least 30 years without evidence of association with Legionnaires’ disease cases. All isolates belonged to one of three sequence types (STs), ST27 (*n*=81), ST68 (*n*=122) and ST87 (*n*=32), all three of which have been recovered from Legionnaires’ disease patients previously. Pairwise single nucleotide polymorphism differences amongst isolates of the same ST were low, ranging from 0 to 19 in ST27, from 0 to 30 in ST68 and from 0 to 7 in ST87, and no homologous recombination was observed in any lineage. However, there was evidence of horizontal transfer of a plasmid, which was found in all ST87 isolates and only one ST68 isolate. A single ST was found in 10/13 sampled locations, and isolates of each ST were also more similar to those from the same location compared with those from different locations, demonstrating spatial structuring of the population within the water system. These findings provide the first insights into the diversity and genomic evolution of a *L. pneumophila* population within a complex water system not associated with disease.

## Data Summary

All raw sequence data has been deposited in the European Nucleotide Archive under the study accession number PRJEB12239/ERP013693 and individual run accession numbers are also available in Table S1 (available with the online version of this article).

**Impact Statement**

In this work, we used genome data to investigate the population diversity of *Legionella pneumophila* within the water system of a large occupational building. The building is thought to have been colonized by *L. pneumophila* for over 30 years. While previous studies have analysed the genomic diversity of *L. pneumophila* in water systems associated with cases of Legionnaires’ disease, this is believed to be the first study to investigate a water system that has no known association with disease cases using high-resolution genomic analysis. We show that the water system has been colonized by three distinct strains, or sequence types (STs), with low diversity amongst isolates of each ST. There is evidence for spatial structuring of the population throughout the water system, suggesting that the building has been colonized a limited number of times (perhaps only once) by each ST. Finally, we show that analysis of a single water sample will usually provide representation of the different STs present in the immediate vicinity, but will likely fail to capture the full spectrum of genomic variation needed for high-resolution analysis. This has implications for future environmental investigations, and suggests that deep sampling and typing of multiple isolates are required.

## Introduction

*Legionella pneumophila* is a Gram-negative bacterium found in fresh water and soil environments as a facultative parasite of protozoa [1]. It also colonizes artificial water systems from which humans can become infected, usually via inhalation of contaminated aerosols [2]. Infection can result in either a mild flu-like illness (Pontiac fever) or a severe, life-threatening pneumonia (Legionnaires’ disease). Common sources of infection include the hot- and cold-water systems of large buildings, decorative fountains, spa pools, humidifiers and cooling towers [3–9].

Several factors are thought to enhance colonization of water systems by *L. pneumophila*, including temperature, sediment accumulation and the presence of other microbiota [10]. Furthermore, water systems of large buildings (e.g. hospitals, hotels and offices) are particularly at risk of colonization due to the complexity of the pipe networks, the large number of outlets and the possibility of dead-legs and blind-ends harbouring stagnant water [11]. Several control measures are commonly used, including management and maintenance of the water system and associated facilities, temperature regulation and chemical biocides. Pasteurization, super-chlorination and point-of-use filtration are also employed as emergency control measures. However, it is recognized that total eradication is very difficult once a building has been colonized [12]. Thus, the aim of control measures is to control the bacterial load of *L. pneumophila* in a water system, which rather than the presence of colonization *per se* is thought to be the most important indicator of disease risk.

A number of studies have demonstrated colonization of hospital water systems with *L. pneumophila* over many years despite implementation of control measures [13, 14]. Some of these studies have also shown persistence of the same strain [15–17] using typing techniques such as pulsed field gel electrophoresis (PFGE) and sequence-based typing (SBT) [18–20], which is analogous to multi-locus sequence typing (MLST). However, by using whole-genome sequencing (WGS), it is now possible to examine population diversity at a much greater resolution. Only two studies have so far extensively examined the genomic diversity of *L. pneumophila* populations within individual water systems and, in both studies, a hospital setting associated with nosocomial Legionnaires’ disease cases over several years was investigated [21, 22]. Approximately 40 isolates from hospital water systems were analysed in each study, demonstrating substantial genomic diversity amongst sequence type (ST) 1 isolates. The studies showed that analyses of multiple environmental isolates from a water system, even of the same ST, are required for a robust comparison with potentially linked clinical isolates in outbreak scenarios.

To our knowledge, the genomic diversity of *L. pneumophila* from artificial water systems that have no known association with Legionnaires’ disease cases has yet to be investigated, and may provide further clues as to how *L. pneumophila* populations evolve in the environment, and how and why in certain instances this bacterium emerges as a human pathogen. Here, we used WGS to analyse 235 isolates obtained from the water system of a large occupational building in the UK that had evidence of colonization for over 30 years, but which has never been associated with disease.

## Methods

### Study setting

The occupational building under study was occupied by >1000 staff. The water system was complex and contained numerous water outlets with variable usage. The building was thought to have been colonized by *L. pneumophila* for over 30 years and serogroup (sg) 3 was thought to have been detected in 1987, although these observations were unconfirmed. In recent years, the water system had been chlorinated annually and each calorifier was pasteurized twice a year. Outlets were flushed weekly.

### Sample collection and identification

Twenty-eight water samples (1 l) were obtained from hot and cold outlets in 13 locations of the occupational building between 15 January and 26 February 2013. Water samples were processed according to a method adapted from ISO 11731 : 1998 part1. An aliquot of each sample (0.1 ml) was cultured on glycine vancomycin polymyxin cycloheximide (GVPC) medium (Oxoid) directly, and the remaining sample was concentrated by membrane filtration using a 0.2 µm polyethersulfone filter (Pall) and the bacteria resuspended in 1 ml Pages saline. Sample concentrates (0.1 ml) were cultured on GVPC either directly (untreated) or after heat (30 min at 50 °C) or acid (0.2 mol HCl-KCl l^−1^, pH 2.2 for 5 min) treatments. Plates were incubated at 36±1 °C for 10 days. Colonies with a ‘ground-glass’ appearance and a requirement for l-cysteine, which are both indicative of the genus *Legionella*, were screened by latex agglutination test according to the manufacturer’s instructions (Oxoid) to preliminarily identify *L. pneumophila* isolates. The theoretical limit of detection is 10 c.f.u. l^−1^ for untreated and heat-treated samples, and 20 c.f.u. l^−1^ for acid-treated samples.

### Identification confirmation and initial serogrouping

Isolates preliminarily identified by latex agglutination as *L. pneumophila* were tested by PCR as described elsewhere [23] to confirm the identification and to determine whether they were carrying the sg 1 marker, *wzm* [24].

### Culture, DNA extraction and WGS

*L. pneumophila* isolates were bead-stored at −80 °C and later grown at 37 °C on buffered charcoal yeast extract with α-ketoglutarate (BCYEK) agar (Oxoid) for 48–72 h. DNA was manually extracted using a Wizard kit (Promega). DNA was eluted in 1× Tris-EDTA (TE) buffer (pH 8.0) and quantified using GloMax (Promega). WGS was performed on the Illumina X10 platform at the Wellcome Sanger Institute (Hinxton, UK) using 150 bp paired-end reads. All raw sequence data has been deposited in the European Nucleotide Archive under the study accession number PRJEB12239/ERP013693 and individual run accession numbers are also available in Table S1.

### *De novo* assembly

Assemblies were produced using VelvetOptimiser and Velvet [25]. An assembly improvement step was then applied to the assembly with the highest N50 value (i.e. the minimum contig length needed to cover 50 % of the total assembly length), the contigs were scaffolded using sspace [26] and sequence gaps filled with GapFiller [27]. Assembly statistics are provided in Table S1.

### *In silico* determination of ST and sg 1/non-sg 1

The STs of the isolates, as defined by the *L. pneumophila* SBT scheme [18–20], were determined using MLSTcheck [28]. blastn [29] was used to identify the presence/absence of the *wzm* gene, a marker of sg 1 [24], in the assemblies.

### Serogrouping and mAb typing

A representative isolate from each ST (ST27 – RR13000067, ST68 – RR13000123, ST87 – RR13000058) was fully characterized phenotypically using the Dresden panel of mAbs to determine the sg and subgroup [30].

### Mapping and phylogenetic analysis

To reconstruct a phylogenetic tree of all 235 isolates from the occupational building, sequence reads were mapped to the Corby reference genome [31] using bwa [32]. Mapping statistics are provided in Table S1. To reconstruct a phylogenetic tree of each ST separately and achieve higher resolution, sequence reads from each ST were also mapped to a *de novo* assembly of a newly sequenced isolate of the same ST (ST27 – RR13000062, ST68 – RR13000200, ST87 – RR13000070). Previously published sequence data from isolates belonging to ST27, ST68 and ST87 were also included [33–35]. Single nucleotide polymorphisms (SNPs) were called using an in-house pipeline, comprising SAMtools, mpileup and BCFtools [36], and pseudo-genome alignments generated. Putative recombined regions were identified from alignments of the single STs using Gubbins [37]. Maximum-likelihood trees were reconstructed using the variable sites with RAxML using a general time reversible (GTR) model with a gamma correction for site rate variation [38]. One hundred bootstrap replicates were performed. Pairwise SNP differences between isolates were also calculated from these alignments. Panito (available at https://github.com/sanger-pathogens/panito) was used to determine the average nucleotide identity (ANI) between representatives of each of the three STs (the same as those used for the mapping references) from the pseudo-genome alignments generated by mapping to the Corby genome.

### Dating analysis

The maximum-likelihood trees of each of the three STs comprising the isolates from the occupational building were rooted using outgroup isolates of the same ST (ST27 – WX2011046 [35], ST68 – EUL 153/EUL 158 [34], ST87 – LC6677 [33]). Accelerated transformation parsimony was used to reconstruct the number of SNPs that have occurred on each branch of the tree, meaning that SNPs were inferred to have occurred as early as possible. The time taken for those SNPs to occur was then estimated for each branch using the published evolutionary rates for the ST37 [39] and ST578 [40] lineages. The time taken for each root-to-tip distance of the tree to have occurred was then determined by summing the times for the constituent branches, and the median of these values was calculated.

### Identification of core and accessory genes

Assemblies were annotated using Prokka v1.5 [41]. Roary [42] was used to identify core and accessory genes amongst the 235 assemblies using an identity threshold (blastp) of 95 %.

## Results

### Identification of three STs in the water system of a large occupational building

We used WGS to analyse 235 *L. pneumophila* isolates obtained from the water system of a large occupational building in the UK (Table S1). Detection of the *wzm* gene, a marker of *L. pneumophila* sg 1, in the genome assemblies and also by conventional PCR, revealed that 81/235 (34.5 %) isolates belonged to sg 1 and 154/235 (65.5 %) belonged to sg 2–14. Using the SBT scheme [18–20], analogous to MLST, all 81 sg 1 isolates were subsequently typed (*in silico*) as ST27, while 122/154 (79.2 %) and 32/154 (20.8 %) sg 2–14 isolates were identified as ST68 and ST87, respectively. Although the SBT gene, *mompS*, is known to be duplicated in some *L. pneumophila* genomes resulting in ambiguous allele calls with short-read data, only one *mompS* variant was detected in all 235 genomes. Despite being of different sgs, ST27 and ST68 are triple-locus variants (TLVs), meaning that they share 4/7 of the SBT alleles (*flaA*, *asd*, *mompS*, *proA*). Representative isolates from ST68 and ST87 were selected for further *in vitro* serogrouping, as well as an isolate from ST27 for sg 1 subgrouping by mAb. The ST68 isolate (RR13000123) was typed as sg 6, the ST87 isolate (RR13000058) as sg 3 and the ST27 (sg 1) isolate (RR13000067) as ‘Knoxville’ subgroup.

We searched the *L. pneumophila* SBT database (http://www.hpa-bioinformatics.org.uk/legionella/legionella_sbt/php/sbt_homepage.php) for submitted isolates belonging to the same STs as those we identified in the occupational building, in order to observe the relative frequency of their isolation in clinical and environmental samples, and their geographical distribution. As of 22 March 2018, 26 ST27 isolates had been submitted to the database, including 18 clinical and 8 environmental isolates (21 from the UK, 1 from the USA and 4 from an unknown country of origin). A total of 68 ST68s had been submitted to the database (28 clinical and 39 environmental isolates and 1 from an unstated source) from several European countries, Japan and Canada. Finally, the SBT database contained details of 61 ST87 isolates (18 clinical and 43 environmental isolates), and these were also submitted from several European countries, as well as Russia, Israel and Japan. However, these results are subject to bias, since the SBT database is limited to submission based upon individual researcher requirements and likely only represents a small proportion of sampled isolates.

### Isolates belonging to STs 27, 68 and 87 are highly clonal

**Fig. 1.**
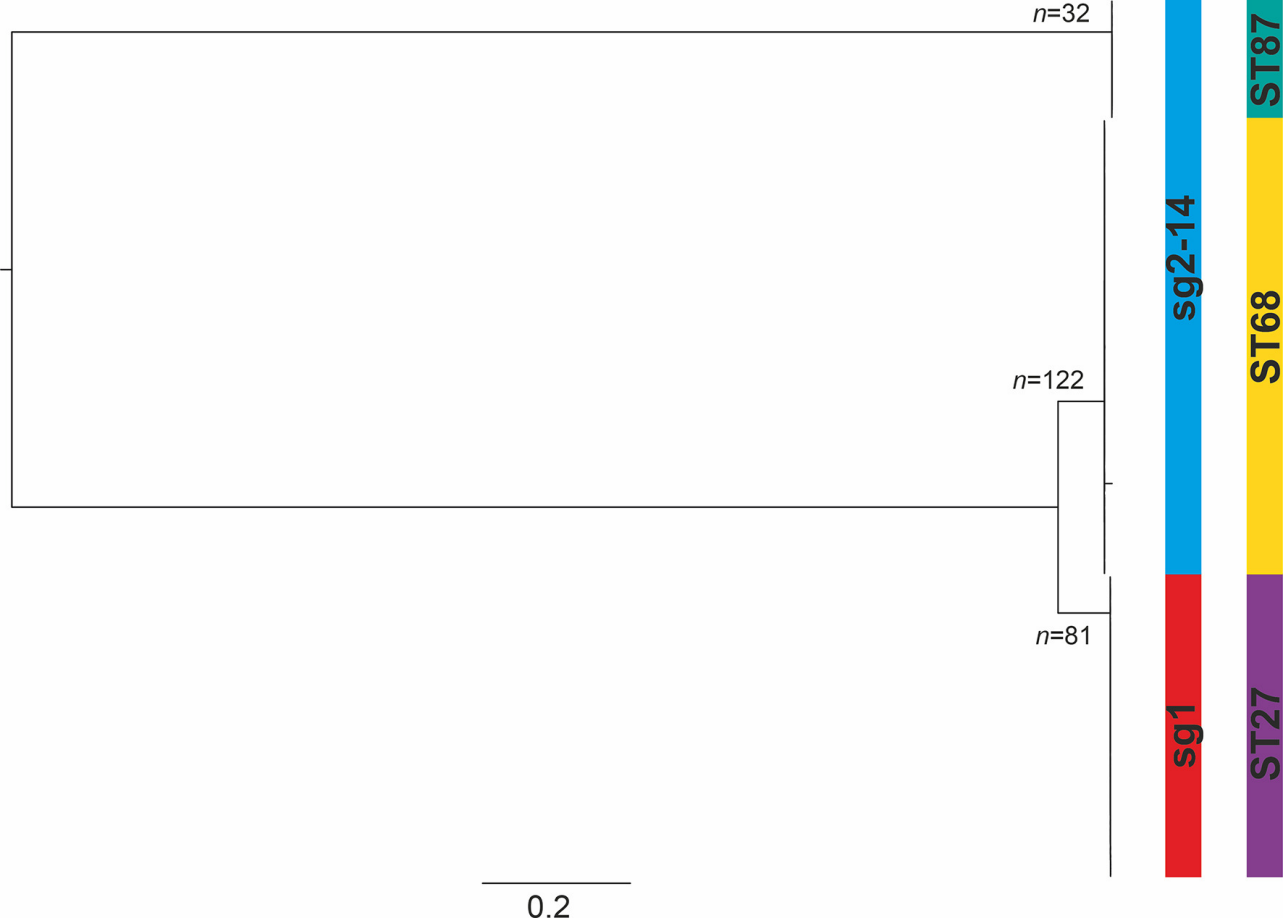
Maximum-likelihood tree of 235 *L. pneumophila* isolates obtained from the occupational building, reconstructed after mapping sequence reads to the Corby reference genome. The columns show the sg, as inferred from the presence/absence of the *wzm* gene, and the ST. Bar, number of SNPs per variable site in the genome alignment.

**Fig. 2.**
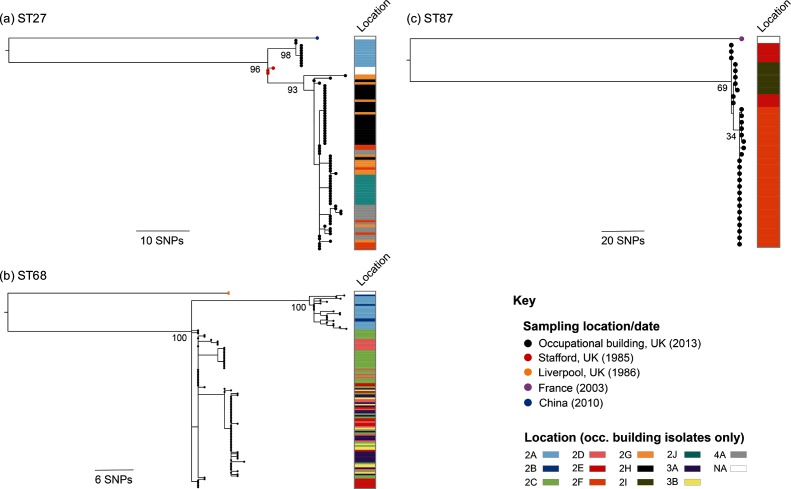
Maximum-likelihood trees of 85 ST27 (a), 124 ST68 (b) and 33 ST87 (c) isolates obtained from the occupational building and elsewhere, reconstructed after mapping sequence reads to ST-specific reference genomes and the removal of recombined regions from the alignments. The trees were rooted using isolates obtained from elsewhere as outgroups. The tips of the trees (circles) are coloured by the general sampling location and date, and the columns show the locations of isolation in the occupational building (if applicable). Bootstrap values, derived from 100 resamples, are shown for major nodes.

The phylogenetic tree of all 235 isolates sampled from the occupational building, reconstructed by mapping sequence reads to the Corby reference genome [31], demonstrated that ST27 and ST68 isolates (the TLVs) are closely related and both more distantly related to ST87 (Fig. 1). The number of SNPs between a representative isolate from each of ST27 and ST68 is 3809 (ANI=99.8 %), while the numbers of SNPs between representatives of STs 27 and 68 with ST87 are 30 560 (ANI=98.8 %) and 35 604 (ANI=98.6 %), respectively. To analyse each ST at higher resolution, isolates were also mapped to a reference genome of the same ST. We searched for regions of recombination in each ST-specific alignment using Gubbins [37]. None were found in either of the ST27 or ST87 lineages, and only a 12 bp region that introduced six SNPs was identified as a putative recombined region in the ST68 lineage. Further investigation revealed this to be a result of mis-mapping, but nevertheless the region was removed from the alignment. Pairwise SNP differences were subsequently calculated from the alignments, revealing a range of 0–19 SNPs in ST27, 0–30 SNPs in ST68 and 0–7 SNPs in ST87. Thus, isolates from each ST were shown to be highly clonal. Phylogenetic trees of each of the three STs also demonstrated the existence of two major subclades in each ST within the occupational building (Fig. 2a–c).

Analysing the diversity within and between the three STs in terms of pan-genome content showed ST87 genomes contained a higher number of genes (range 3269–3284, mean 3274) than those belonging to ST27 (range 3069–3075, mean 3071) and ST68 (range 3003–3194, mean 3009). The total number of genes found across all 235 genomes was 4129, and 2286 ‘core’ genes were found in all 235 genomes. A total of 75 ‘accessory’ genes were specific to ST27 (i.e. present in all ST27 genomes and absent in all others), 18 to ST68 and 666 to ST87, reflecting the high divergence of ST87 relative to STs 27 and 68. On average, over 98.5 % of genes found in each genome were found in all genomes of the same ST, demonstrating minimal variation of accessory genes within STs.

**Fig. 3.**
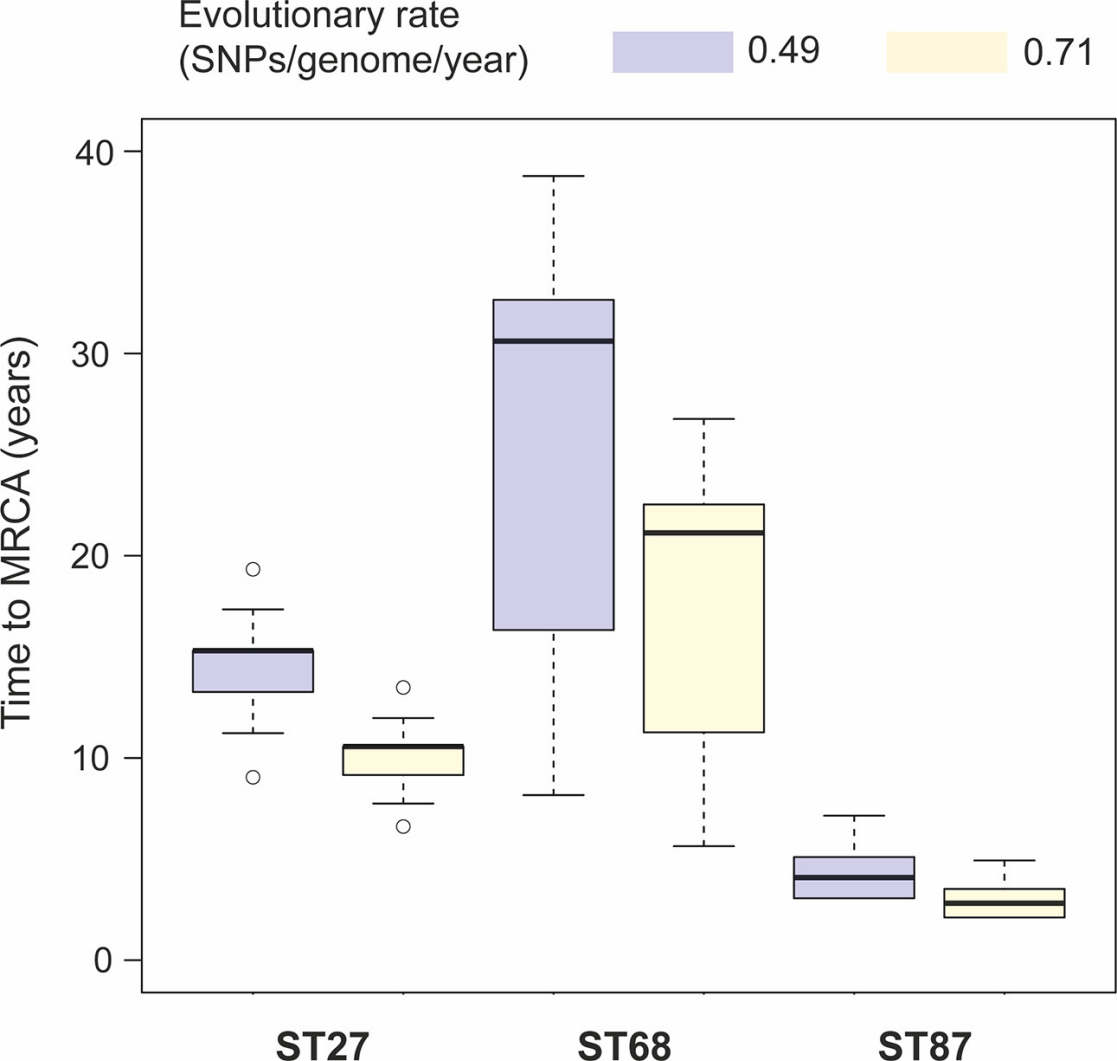
Boxplots showing the estimated time since the MRCA across all root-to-tip distances for isolates belonging to the STs 27, 68 and 87 from the occupational building. Estimates are provided given two different evolutionary rates previously inferred.

Next, we estimated how long ago the most recent common ancestor (MRCA) of each ST in the water system existed, potentially representing the time of introduction of these STs. Since the evolutionary rate of *L. pneumophila* likely varies between lineages and there are no published rates for the three STs under study, we used those that were previously inferred for ST37 [39] and ST578 [40]. Our median estimates based on evolutionary rates of 0.71 SNPs per genome per year (ST37) and 0.49 SNPs per genome per year (ST578) are 10.6/15.3 years for ST27, 21.1/30.6 years for ST68 and 2.8/4.1 years for ST87 (Fig. 3).

### High similarity between ST27 isolates from the occupational building and the 1985 Stafford District General Hospital outbreak

We used previously published *L. pneumophila* genomes belonging to STs 27, 68 and 87 to contextualize the isolates from the occupational building. Genomes from four ST27 isolates were used, including three clinical isolates (EUL 126, EUL 127, EUL 128) recovered from patients associated with the 1985 Stafford District General Hospital outbreak [34] and one isolate (WX2011046) recovered from a cooling tower in China in 2010 [35]. By mapping the sequence reads of these isolates to our ST27 reference genome and removing regions of recombination from the alignment, we found 50–56 SNPs between the Chinese isolate and any of the ST27 isolates from the occupational building. However, 5–14 SNPs were found between the Stafford District General Hospital isolates and the building isolates. Two of the Stafford District General Hospital isolates are positioned on the tree node representing the MRCA of the ST27 isolates from the building (Fig. 2a). Additionally, no recombination regions differentiate the building isolates from the Stafford District General Hospital isolates. This suggests that the water system of the occupational building may have been colonized by a closely related ST27 strain to that attributed to causing the hospital outbreak. We also analysed a further two ST68 genomes (EUL 153, EUL158) [34], which we found to be 34–53 SNPs different to the ST68 isolates in the water system after the removal of recombination regions, as well as one ST87 genome (LC6677) [33], which differed by 89–91 SNPs from the ST87 isolates in the water system.

### Spatial structuring of *L. pneumophila* within the water system

**Fig. 4.**
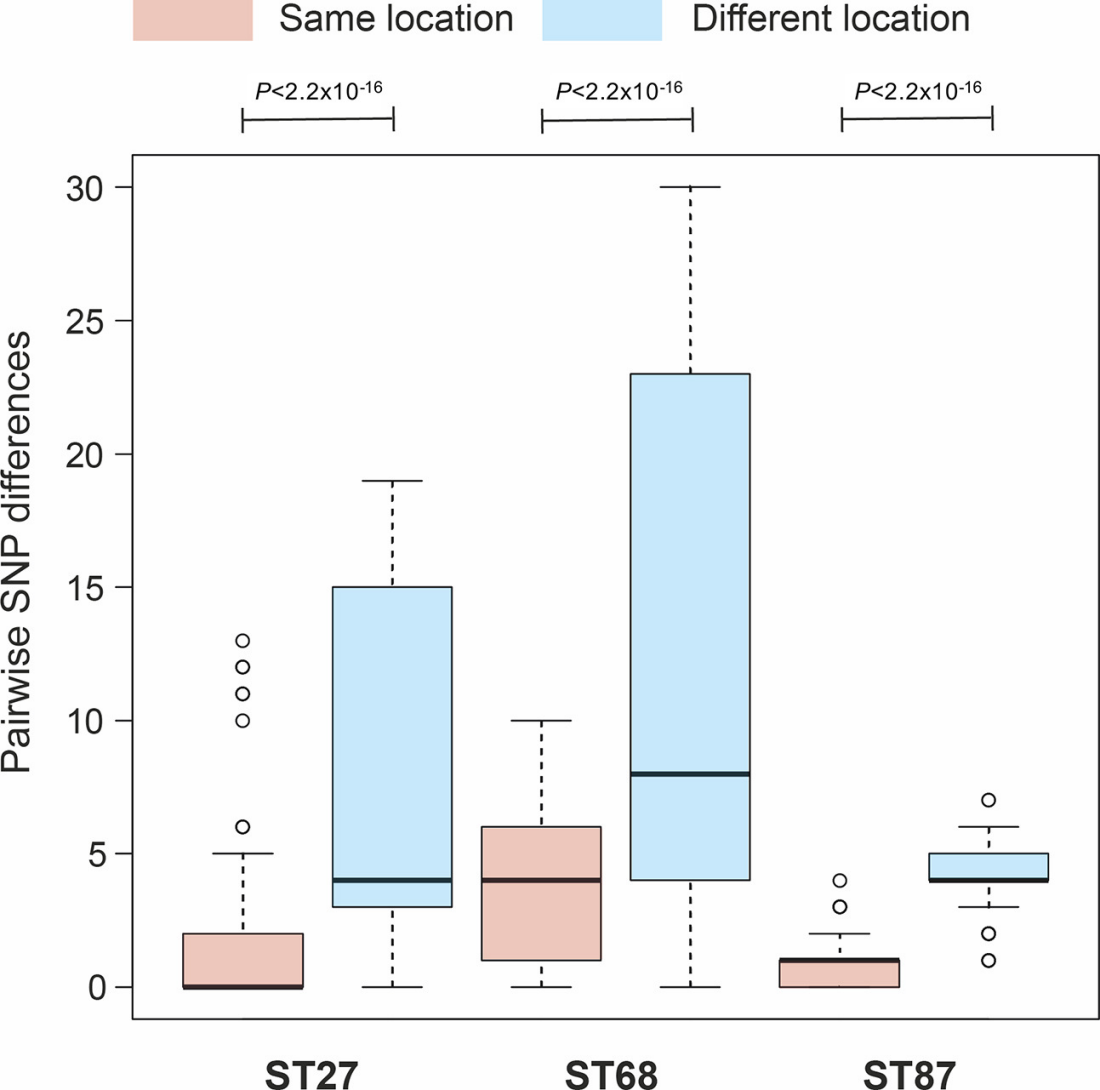
Boxplots showing pairwise SNP differences between isolates from each of the STs 27, 68 and 87 that were sampled from the same and different locations in the occupational building. *P* values were calculated using the Mann–Whitney U-test.

In 10/13 locations in the occupational building from where the *L. pneumophila* isolates were recovered, only a single ST was isolated (Table 1). This suggests that the STs may be spatially structured throughout the water system and uncommonly found together, although this may also reflect a bias of testing a limited number of isolates per sample. Amongst the three locations in which more than one ST was observed, one location contained ST27 and ST68, another contained ST27 and ST87, and the other ST68 and ST87; thus, all combinations of ST pairs were observed. However, all three STs were never found together. We also observed phylogenetic clustering of same-ST isolates by location (Fig. 2a–c) and pairwise SNP differences between isolates of the same ST sampled from the same location are significantly lower than those sampled from different locations (Mann-Whitney U test; *P* values <2.2×10^−16^) (Fig. 4). This further demonstrates spatial structuring of the population throughout the water system and the possibility for local evolution of strains.

**Table 1.** Diversity of isolates in individual locations and water samples The SNPs were calculated after removal of putative recombined regions (only one region in the ST68 lineage). na, Not applicable.

**Location**	**Sample no.**	**STs (and no. of isolates) per sample**	**Range (and mean no.) of SNPs between all same-ST isolates in the sample**	**Range (and mean no.) of SNPs between all same-ST isolates in the location**
2A	1	ST27 (6)	0 SNPs between all	ST27 : 0–1 (mean 0.3) ST68 : 0–9 (mean 4.3)
		ST68 (8)	0–9 (mean 5.2)	
	2	ST27 (5)	0–1 (mean 0.6)	
		ST68 (10)	0–8 (mean 3.6)	
2B	3	ST68 (4)	1–9 (mean 4.8)	ST68 : 1–9 (mean 4.8)
2C	4	ST68 (14)	0–6 (mean 2.8)	ST68 : 0–10 (mean 4.5)
	5	ST68 (4)	0–5 (mean 3.2)	
	6	ST68 (15)	0–10 (mean 3.4)	
	7	ST68 (2)	4	
	8	ST68 (1)	na	
2D	9	ST68 (10)	0–7 (mean 2.6)	ST68 : 0–9 (mean 4.3)
	10	ST68 (8)	0 SNPs between all	
2E	11	ST68 (8)	0–3 (mean 1.1)	ST68 : 0–9 (mean 3.8) ST87 : 0–1 (mean 0.6)
		ST87 (5)	0–1 (mean 0.6)	
	12	ST68 (7)	0–1 (mean 0.3)	
2F	13	ST27 (4)	0–4 (mean 2)	ST27 : 0–4 (mean 1.5) ST87 : 0–4 (mean 0.8)
		ST87 (11)	0–3 (mean 1.2)	
	14	ST27 (4)	0–2 (mean 1)	
		ST87 (11)	0–2 (mean 0.4)	
2G	15	ST27 (1)	na	ST27 : 0–13 (mean 4.2)
	16	ST27 (3)	1–6 (mean 4)	
	17	ST27 (9)	0–12 (mean 4.6)	
2H	18	ST27 (15)	0 SNPs between all	ST27 : 0–3 (mean 0.4)
	19	ST27 (2)	3	
	20	ST27 (7)	0 SNPs between all	
2I	21	ST87 (5)	0–1 (mean 0.4)	ST87 : 0–1 (mean 0.4)
2J	22	ST27 (11)	0 SNPs between all	ST27 : 0 SNPS between all
	23	ST27 (1)	na	
3A	24	ST68 (6)	0–2 (mean 0.7)	ST68: 0–3 (mean 0.9)
	25	ST68 (15)	0–3 (mean 1.0)	
3B	26	ST68 (6)	0–2 (mean 0.7)	ST68: 0–2 (mean 0.4)
	27	ST68 (4)	0 SNPs between all	
4A	28	ST27 (13)	0–6 (mean 2.4)	ST27 : 0–6 (mean 2.4)

We obtained two or more water samples from 10 locations, including the 3 locations from which 2 STs were recovered, and could test whether isolates recovered from a single water sample provide representation of the overall diversity in the area. Indeed, in 9/10 locations, the same ST(s) were found in the different samples from each location, suggesting that one water sample usually provides full representation at the ST level (Table 1). Only in one location (2E), both ST68 and ST87 isolates were recovered from one water sample, while only ST68 isolates were recovered from another (albeit taken from a different outlet). However, we also found that in 24/25 samples that were taken from these 10 locations, there was lower SNP variation (i.e. fewer variable positions) amongst same-ST isolates than the amount observed in the area overall. This may reflect spatial structuring of the isolates by water outlets, although we do not have precise information on the sampled outlets.

### Evidence for plasmid transfer between the STs

Although no homologous recombination could be detected in any of the three ST lineages found in the occupational building, we investigated whether there was evidence of sharing of accessory genes between the different STs. We searched for evidence of recent gene transfer between STs by identifying genes present in all genomes of one ST, but <95 % of genomes of another. Most significantly, we found 193 genes that were present in all ST87 genomes but only a subset of ST68 genomes, and 174/193 of these were found in only one ST68 genome (RR13000178). These genes were located on a single 170 kb contig in the ST87 reference assembly, and their functional annotations (e.g. presence of *tra* genes) are suggestive of a plasmid, which may therefore have been horizontally transferred to the ST68 isolate. A blastn search of this sequence against the National Center for Biotechnology Information nr database found no matches across the full length, although a 38 kb region containing a F-type IVA secretion system did match the sequence of a *Legionella longbeachae* plasmid (GenBank accession no. CP020895.2) with 98 % nucleotide identity. The ST87 contig also contained numerous heavy metal resistance genes, including the copper-resistance gene, *copA*, and the cobalt/zinc/cadmium efflux *helABC* operon. The ST68 isolate was also recovered from a location (2E) from which ST87 isolates were sampled.

## Discussion

Here, we present the largest genomic study to date of environmental *L. pneumophila* isolates from a water system of an occupational building. We found co-existence of three highly clonal populations of isolates belonging to STs 27, 68 and 87. All three STs have previously been recovered from Legionnaires’ disease patients (http://www.hpa-bioinformatics.org.uk/legionella/legionella_sbt/php/sbt_homepage.php), suggesting that the building isolates have pathogenic capacity. Interestingly, ST27 isolates from the occupational building were found to be highly similar (a minimum difference of five SNPs) to three clinical ST27 isolates recovered from patients associated with a large outbreak of Legionnaires’ disease at Stafford District General Hospital in 1985 that was linked to a cooling system [43]. Since, to our knowledge, the occupational building has never been linked to cases of Legionnaires’ disease, this suggests that the genetic composition of this strain may not have been the crucial factor contributing to the hospital outbreak, but rather other local factors including the method of exposure (contaminated aerosol from air-conditioning), the *L. pneumophila* concentration and/or the immune status of the exposed individuals. Furthermore, the low number of SNPs identified between ST27 isolates from 1985 and 2013 adds to the growing evidence that *L. pneumophila* has a very low evolutionary rate leading to extreme temporal and spatial conservation [39, 40, 44].

We found evidence for spatial structuring of the *L. pneumophila* population within the water system at the ST level and also within STs. This implies that the building has been colonized a limited number of times (perhaps only once) by each of the three STs. Our estimates of the time to the MRCA for each of the three STs range from 2.8–4.1 years for ST87 to 21.1–30.6 years for ST68, suggesting that the building may have been colonized at different times by each of the three STs. However, it could also be that some populations have been subjected to bottlenecks, have different evolutionary rates or have been sampled at different densities, all of which would alter the observed levels of variation and affect the estimates of the time to the MRCA. Indeed, sg 3 was detected in 1987, yet our ST87 isolates obtained in 2013 (which may represent the same strain since one isolate typed as sg 3) were found to be the least diverse of the three STs. It is also intriguing that the three STs were never found all together, neither in a single sample, nor from different samples collected from the same location. While this may be the result of a sampling artefact, it is plausible that co-existence of all three STs in the same location is ecologically improbable due to between-ST competition dynamics. However, this would need to be confirmed using *in vitro* competition experiments. The presence of only one ST in most locations could also explain the lack of recombination observed between the three STs, which may otherwise have been expected given the recombinogenic nature of *L. pneumophila* [39, 40]. Finally, our findings demonstrate that while a single sample will usually provide representation of the different STs present in the immediate vicinity, it will likely fail to capture the full spectrum of SNP-level differences that allow high resolution analysis. This reinforces the need for deep sampling and typing of multiple isolates during environmental investigations.

## Data bibliography

1. Underwood AP, Jones G, Mentasti M, Fry NK, Harrison TG. European Nucleotide Archive, https://www.ebi.ac.uk/ena/data/view/ERR315653 – accession number ERR315653 (2013).
2. David S, Mentasti M, Tewolde R, Aslett M, Harris SR *et al*. European Nucleotide Archive, https://www.ebi.ac.uk/ena/data/view/PRJEB1828 – accession numbers ERR376691, ERR376761, ERR376699, ERR376775 and ERR376780 (2016).
3. Qin T, Zhang W, Liu W, Zhou H, Ren H *et al*. European Nucleotide Archive, https://www.ebi.ac.uk/ena/data/view/LBAK01000000 – accession number LBAK01000000 (2016).

## Supplementary Data

Supplementary File 1
